# Expression and Regulation of *pde6h* by Thyroid Hormone During Metamorphosis in *Paralichthys olivaceus*

**DOI:** 10.3389/fphys.2020.00244

**Published:** 2020-04-02

**Authors:** Yuejuan Cheng, Jiaqian Xu, Yuanshuai Fu, Nisha He

**Affiliations:** ^1^Key Laboratory of Freshwater Aquatic Genetic Resources, Ministry of Agriculture, Shanghai Ocean University, Shanghai, China; ^2^Key Laboratory of Exploration and Utilization of Aquatic Genetic Resources, Ministry of Education, Shanghai Ocean University, Shanghai, China; ^3^Shanghai Collaborative Innovation for Aquatic Animal Genetics and Breeding, Shanghai Ocean University, Shanghai, China; ^4^State Key Laboratory of Biocatalysis and Enzyme Engineering, Hubei University, Wuhan, China

**Keywords:** *Paralichthys olivaceus*, metamorphosis, thyroid hormone, *pde6h*, dual-luciferase

## Abstract

*PDE6H* is a cone cell-specific inhibitory subunit that plays a critical role in the adaptation of the photosensitive system to bright and dark phases of the light environment. Thyroid hormone (TH) is one of the most important factors that control development and metabolism in animals, composed mainly of triiodothyronine (T3), and thyroxine (T4). TH also plays a key role in the metamorphosis of the flounder (*Paralichthys olivaceus*), wherein exogenous TH can accelerate the behavioral changes of larvae from the pelagic to benthic type accompanying changes in the light environment from bright to dark. In this study, transcriptional analysis showed that *pde6h* is expressed in adult eye, that its expression peaks at the climax of metamorphosis, and that it can be significantly up-regulated to the highest level by exogenous T4 in the early stages of metamorphosis but is inhibited by thiourea (TU). The rescue experiment showed that metamorphic inhibition of larvae and expression inhibition of *pde6h* gene in TU groups can be rescued by removing TU. Further, dual-luciferase reporter assay indicated the putative regulatory effect of TH on *pde6h* expression, mediated directly on the gene promoter by the TRαA gene. Together, we speculated that TH may control physiological adaptation of the photosensitive system to light changes during metamorphosis by acting directly on *pde6h*. This study can help us further study the physiological function of *pde6h* during flounder metamorphosis in the future.

## Introduction

Phosphodiesterases 6 is a photoreceptor cell-specific subfamily of phosphodiesterases (PDEs), consisting of α, β, and α’ catalytic subunits encoded by the *PDE6A*, *PDE6B*, and *PDE6C* genes, and γ and γ’ inhibitory subunits encoded by the *PDE6G* and *PDE6H* genes, respectively (Cote, 2004; Conti and Beavo, 2007). PDE6s are distinctively expressed in vertebrate rod and cone photoreceptor cells. Rods express the *PDE6A* and *PDE6B* genes, which form a catalytic heterodimer, and the *PDE6G* inhibitory subunit gene, whereas cones express *PDE6C*, which forms a catalytic homodimer, and the *PDE6H* inhibitory subunit gene (Cote, 2004; Larhammar et al., 2009; Lagman et al., 2016). The PDE6 enzymes include two catalytic subunit proteins, which have two GAF domains and one catalytic domain, and two accessory inhibitory subunits (Guo et al., 2006; Conti and Beavo, 2007). Under dark conditions, the accessory inhibitory subunits interact with a GAF domain and the catalytic domain of the catalytic subunits and thus block PDE6 activity (Guo et al., 2006). In contrast, under light conditions, photon-activated opsins promote a GTP molecule to replace GDP at the active site of the α subunit of the heterotrimeric G-protein transducin, thereby resulting in the dissociation of transducin into an activated α subunit and a heterodimer of β and γ subunits (Lagman et al., 2016). The α subunit then activates PDE6, which hydrolyzes cGMP into GMP. The reduction in cGMP levels leads to the closure of cyclic nucleotide-gated channels and results in hyperpolarization of the photoreceptor cell (Arshavsky et al., 2002; Zhang et al., 2012; Lagman et al., 2016). Despite the cloning of cone cell-specific inhibitory subunit *PDE6H* more than 20 years ago, its regulation in vertebrate development remains unknown.

Flounder (*Paralichthys olivaceus*), an important marine fish, undergoes a dramatic metamorphosis from larval to juvenile stage (Inui and Miwa, 1985). The metamorphosis is accompanied by drastic morphological, physiological, and behavioral changes. Particularly, the right eye moves to the left and the lifestyle changes from pelagic to benthic, which may be closely related to the development of a retinal photosensitive system. It is known that exogenous thyroid hormone (TH) can accelerate the metamorphic process of *P. olivaceus*, while thiourea (TU), a TH synthesis inhibitor, blocks metamorphosis (Schreiber and Specker, 1998). TH includes two main hormones, namely triiodothyronine (T3) and thyroxine (T4). T4 is a pro-hormone, whereas T3 is the hormone that binds to thyroid hormone receptor (TR) *in vivo* (Harvey and Williams, 2002; Carvalho and Dupuy, 2017). TRs are nuclear receptors comprising two main classes, alpha, and beta (Flamant et al., 2006). TRs act as transcription factors, ultimately affecting the regulation of gene expression by binding to T3 (Flamant et al., 2006; Ortiga-Carvalho et al., 2014).

Although some studies in recent years have shown that TH regulates cone photoreceptor differentiation in the retinas (Kelley et al., 1995; Applebury et al., 2000; Ng et al., 2001; Mader and Cameron, 2006; Roberts et al., 2006; Srinivas et al., 2006; Trimarchi et al., 2008), there is surprisingly little known about the relationship between TH and visual signal transduction in vertebrate development. In this study, we analyzed the expression of *pde6h* gene during metamorphosis and in adult tissues and identified the regulatory relationship between TH and *pde6h* in *P. olivaceus*. The results showed that *pde6h* was mainly expressed in adult eye, was highly expressed at the metamorphic climax, and can be directly regulated by T3 binding to TRαA. This study will fulfill a need to address the currently deficient understanding of the expression and regulation of *pde6h* in the flounder and its roles in development.

## Materials and Methods

### Ethics Statement

Our study was performed in strict accordance with Laboratory Animals—Guidelines for ethical review of animal welfare of China (GB/T 35892-2018). All experimental procedures were approved by the Animal Ethics Committee of Shanghai Ocean University (SHOU-DW-2017-039).

### Fish Samples

Flounder (*P. olivaceus*) were collected from the Beidaihe Central Experiment Station (Chinese Academy of Fishery Sciences, Hebei Province, China). Larvae at 15 days post-hatching (dph) were randomly divided into three groups (2000 fish per tank): the NC group (normal control) was cultured with natural seawater, the TH group was exposed to seawater with 130 nM of exogenous T4 (Sangon, Shanghai), and the TU group was exposed to seawater containing 30 mg/L of exogenous TU (thiourea; Sangon, Shanghai) (Inui and Miwa, 1985). These three groups of larvae were cultured with *Artemia nauplii* from 15 dph till the end of the experiment. According to Minami (1982) (Minami, 1982), whole larvae (*n* = 6 pools in each group, 3 specimens/pool) from NC, TH, and TU groups were periodically collected at 16 dph (Early metamorphosis I, the stage prior to the start of eye migration), 21 dph (Early metamorphosis Ï, when the right eye has started to shift and six coronal fins begin to elongate), 25 dph (Metaphase metamorphosis I, when the right eye has become visible from the ocular side but has not reached the dorsal midline), 28 dph (Mid-metamorphosis Ï, climax metamorphosis, when the right eye has become visible from the ocular side and reached the dorsal midline and coronal fins assume the greatest length), 31 dph (Late metamorphosis I, the right eye has just become located on the overhead and starts to move to the left side of the body and coronal fins are significantly shortened), 36 dph (Late metamorphosis Ï, the right eye is on the left side of the body, coronal fins still have remnants, and body surface melanin increases), and 41 dph (juvenile, the right eye has completely moved to the left side of the fish body, the coronal fins disappear, and the pigment is well developed). All larvae and juveniles were anesthetized with MS-222 (3-Aminobenzoic acid ethyl ester methanesulfonate, Sigma-Aldrich) for observation under a microscope, washed with DEPC water, and stored with RNAlater (Invitrogen, Life Technology, Carlsbad, CA, United States) at −80°C.

*Paralichthys olivaceus* adults were firstly anesthetized with MS-222 and were killed by decapitation. Tissues containing the heart, liver, stomach, kidney, brain, gill, muscle, eye, and intestine were then collected by dissecting six fish (*n* = 6) and directly frozen in liquid nitrogen. All samples were stored at −80°C for RNA extraction until subsequent RNA isolation.

### Rescue Experiment

To investigate whether the metamorphosis-inhibited larvae in the TU-treated group can be rescued, TU-treated larvae at 35 dph were divided into three groups: the TU group (TU-treated larvae cultured in seawater containing 30 mg/L TU), the TU + NC group (TU-treated larvae cultured in natural seawater), and the TU + TH group (TU-treated larvae cultured in seawater containing 130 nM T4). These three groups of larvae were cultured until 41 dph. Larvae (*n* = 6 pools in each group, 3 specimens/pool) at 36 dph and 41 dph were anesthetized with MS-222, placed in RNAlater, and stored at −80°C for RNA extraction.

### Quantitative Real-Time PCR

Total RNAs were isolated from the whole body of the collected fish samples using TRIzol Reagent (Invitrogen, Life Technology, Carlsbad, CA, United States) according to the manufacturer’s instructions. Total RNAs were treated with RQ1 RNase-free DNase (Promega, Madison, WI, United States) to remove genomic DNA contamination. RNA integrity was assessed by agarose gel electrophoresis, RNA concentration was examined by NANODROP 2000C spectrophotometer (Thermo, Waltham, MA, United States), and 2.0 > A260/280 ratios > 1.8 was considered for RNA purity.

Total RNAs were reverse-transcribed using a RT-PCR kit (Promega, Madison, WI, United States) according to the manufacturer’s instructions. The qRT-PCR (quantitative real-time PCR) primers (Table 1) were designed using Primer Premier 5.0 software for the *pde6h* gene sequence (GenBank: XM_020083982.1). qRT-PCR was conducted using PowerUp^TM^ SYBR^TM^ Green Master Mix on a CFX96Touch^TM^ Real-Time PCR Detection System (Bio-Rad, United States). The total reaction volume was set to 20 μL, comprising 10 μL PowerUp^TM^ SYBR^TM^ Green Master Mix (Applied Biosystems^TM^, United States), 1 μL cDNA template, forward primer (0.2 μM), and reverse primer (0.2 μM) (Table 1). The PCR conditions used are as follows: initial denaturation for 3.0 min at 94°C, followed by 40 cycles at 94°C for 20 s, and at 60°C for 30 s. Each experiment was repeated twice. Samples were run in parallel with the reference gene β-actin. All melting curves were plotted to confirm amplification specificity, and the corresponding efficiencies (E) of qRT-PCR were found to be 0.90–0.99. The relative mRNA expression was determined using the 2^–ΔΔCT^ method (Schefe, 2006).

**TABLE 1:** Primers used in the study.

**Primer**	**Primer sequence (5′ → 3′)**	**Application**
*TR*α*A*1252-F	*G*GAATTCAATGGAGCCAATGTCCAACAA	*TR*α*A* CDS
*TR*α*A*1252-R	*GG*GGTACCTCACACTTCCTGGTCCTCG	
Pro-*pde6h*-F	GGGGTACCGATTCCACCATTATCCGTCA	*pde6h* promotor
Pro-*pde6h*-R	GGGAGCTCCACTGCGCTGTTTCCGTA	
q-*pde6h*-F	AGTAAGGCACCTAAACCA	qRT-PCR
q-*pde6h*-R	AGGAATACATGAGCGACTA	
β-actin F	GGAAATCGTGCGTGACATTAAG	qRT-PCR
β-actin R	CCTCTGGACAACGGAACCTCT	

*F and R, forward and reverse primers, respectively; qRT-PCR, quantitative real-time PCR. The underline represents restriction enzyme sites.*

### Cell Culture

Flounder embryonic cells (FECs), obtained from the Yellow Sea Fisheries Research Institute, Chinese Academy of Fishery Sciences, were cultured in Dulbecco’s modified Eagle’s medium (DMEM; Gibco, CA, United States) supplemented with 20 mM HEPES, pH 7.5, antibiotics (100 U/mL penicillin and 100 μg/mL streptomycin; Gibco BRL), 15% fetal bovine serum (Gibco), sea perch serum (SPS, 0.5%), and basic fibroblast growth factor (bFGF, 2 ng/mL; Gibco BRL). Cells were maintained at 24°C in an ambient air incubator according to Chen’s method (Chen et al., 2004).

### Dual-Luciferase Assay

A ∼2300 bp sequence upstream to the *pde6h* start codon was selected from NCBI GenBank (GenBank: XM_020083982.1) and used to predict the binding site of TRs according to the known sequences of thyroid-hormone responsive elements [TREs: 5′–(A/G)GGT(C/A/G)A–3′], which bind to TRs (Ortiga-Carvalho et al., 2014). The promoter sequence of *pde6h* was PCR-amplified from *P. olivaceus*, and its mutant sequence was synthesized by a biotechnology company (Sangon Biotech, Shanghai, China). They were cloned into pGL3 basic vector to serve as the reporter, respectively, and pRL-TK served as the control reporter. The full-length coding sequence of TrαA was PCR-amplified from *P. olivaceus* and cloned into p3XFLAG under the CMV promoter to serve as an effector, and triiodothyronine (T3) served as the other effector. The recombinant plasmids and T3 (75 nM) were transfected into FECs in the following six groups: pGL3 basic, Propde6h, Propde6h + T3, Propde6h + CMV: TRαA, Mutant + CMV: TRαA + T3 and Propde6h + CMV: TRαA + T3. After transfection for 24 h, the firefly luciferase (LUC) activity was measured with a dual-luciferase reporter assay system (Promega, United States), wherein the Renilla luciferase (REN) activity served as the internal control to evaluate the transfection efficiency. All cell culture experiments were carried out in triplicate. The primers used are listed in Table 1.

### Statistical Analysis

All of the data on *pde6h* expression are represented as mean ± Standard Error (SEM, *n* = 6). Statistical significance was examined using one- or two-way analysis of variance (ANOVA), followed by Tukey’s post-test using SigmaStat 3.5 software. *P-*values < 0.05 were considered to depict significant differences.

## Results

### Temporal Expression of *pde6h* mRNA During Metamorphosis and in Adult Tissues

**FIGURE 1:**
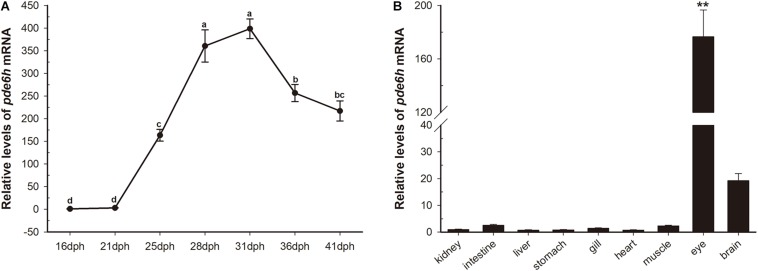
Transient expression of *pde6h* at different stages of larval metamorphosis. **(A)** Expression levels of *pde6h* mRNA during metamorphosis. **(B)** Expression levels of *pde6h* mRNA in the adult tissues. Data are represented as the mean ± SEM (*n* = 6); one-way ANOVA was used to identify significant differences. Different lowercase letters indicate significant differences after Tukey’s post-test (*p* < 0.05), **p < 0.05 represents extremely significant difference compared with other tissues. The *pde6h* levels at 16 dph were used as a reference.

During metamorphosis, *pde6h* levels at 16 dph were used as a reference. As shown in Figure 1A, *pde6h* expression tends to increase and then decrease during metamorphosis and peaks at 31 dph. Specifically, its level rises slowly from 16 dph to 21 dph, increases sharply from 21 dph to 31 dph, and peaks at 31 dph. This is followed by a decrease from 31 dph to 36 dph and a gradual decline from 36 dph to 41 dph. These results show that high expression of *pde6h* gene is synchronous with the peak of metamorphosis.

In adult tissues, *pde6h* levels in the kidney were used as a reference. As shown in Figure 1B, *pde6h* gene was extremely highly expressed in the eye compared to other tissues, indicating that it may be an eye-specific gene in the flounder.

### Effect of TH and TU on *pde6h* mRNA

**FIGURE 2:**
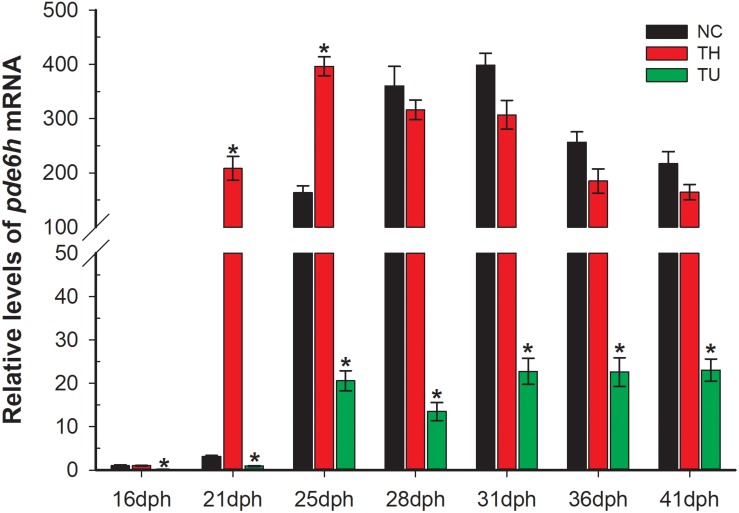
Relative expression of *pde6h* in the NC group, TH group, and TU group during larval metamorphosis. The error bars represent the SEM (*n* = 6); statistical significance was examined by two-way analysis of variance (ANOVA) followed by Tukey’s post-test. *p < 0.05 represents significant difference compared with the NC group at the same development stage.

As shown in Figure 2, exogenous TH and TU significantly affect the expression levels of *pde6h* mRNA. At 21 dph and 25 dph, the level of *pde6h* in the TH group larvae was significantly higher than that of the NC group (*p* < 0.05), while at other time points, there was no significant difference in the *pde6h* levels in the two groups. During metamorphosis, *pde6h* was significantly under-expressed in the TU group larvae compared with in the NC group from 16 dph to 41 dph (*p* < 0.05) and continues to remain at a very low level. These results show that expression of *pde6h* in the three groups of larvae correlates with the presence of exogenous TH regulating larval metamorphosis.

### *pde6h* Expression in the Rescue Experiment of the Metamorphosis-Inhibited Larvae

**FIGURE 3:**
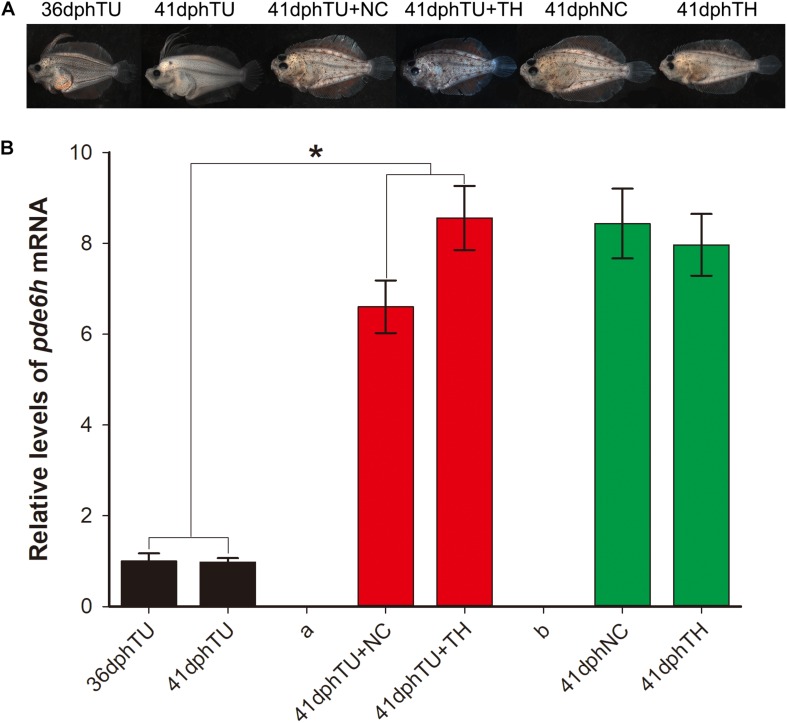
Expression analysis of *pde6h* in the rescue experiment of the TU-inhibited metamorphosis larvae. **(A)** Larvae or juveniles in the rescue experiment of the TU-inhibited metamorphosis larvae. **(B)** Relative levels of *pde6h* upon rescue of the TU-inhibited metamorphosis larvae. The error bars represent the SEM (*n* = 6); statistical significance was detected by two-way analysis of variance (ANOVA) followed by Tukey’s post-test. *p < 0.05 represents significant differences compared to the 36 dph TU group and 41 dph TU group.

In Figure 3A, the metamorphosis-inhibited larvae at 35 dph that were shifted into natural seawater (TU + NC group) and TH seawater (130 nM T4, TU + TH group) and reared for 6 days were successfully rescued at 41 dph. However, the larvae maintained in TU until 41 dph were still found to have impaired metamorphosis. The metamorphosis-completed flounders in the 41 dph NC and 41 dph TH served as control. Alongside, the *pde6h* expression in the different larval groups was detected using qRT-PCR. As shown in Figure 3B, the *pde6h* level in the 41 dph TU + NC and 41 dph TU + TH groups was significantly higher than that of the 35 dph TU (*p* < 0.05) or 41 dph TU (*p* < 0.05) groups, and no significant difference was observed when compared with the 41 dph NC and 41 dph TH groups. The above results indicate that TH might play a key role in regulating *pde6h* expression during metamorphosis.

### Regulation of the *pde6h* Promoter by T3

**FIGURE 4:**
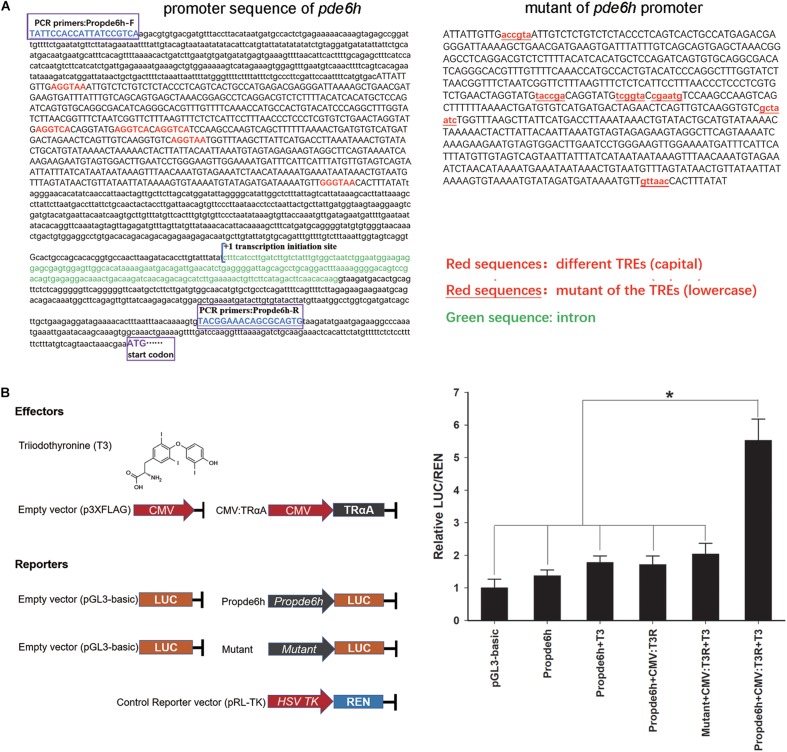
TH binding to TRαA triggers *pde6h* promoter activity. **(A)** Analysis of TREs in the promoter of *pde6h*. **(B)** Dual-luciferase reporter assay of TH binding to TRαA showing the activation of *pde6h* promoter. The error bars represent the SEM (*n* = 3); statistical significance was detected by one-way analysis of variance (ANOVA) followed by Tukey’s post-test. *p < 0.05 represent significant differences compared to the pGL3-basic group. The promoter region upstream of the start codon of *pde6h*, from –2328 to –146, was cloned into the pGL3-basic vector for this analysis.

Analysis of *pde6h* promoter showed the presence of six potential TREs, and 2180 bp of promoter sequence and 712 bp of mutant sequence of the promoter were cloned for dual-luciferase assay (Figure 4A). Dual-luciferase assay was carried out to verify the effect of TRαA and T3 on *pde6h* promoter activity. Our results show that LUC/REN in the Propde6h + CMV:TrαA + T3 group was significantly higher than that in the pGL3-basic (*p* < 0.05), Propde6h (*p* < 0.05), Propde6h + T3 (*p* < 0.05), Propde6h + CMV:TrαA (*p* < 0.05), and Mutant + CMV: TRαA + T3 (*p* < 0.05) group and that there are no significant difference between the pGL3-basic, Propde6h, Propde6h + T3, Propde6h + CMV:TrαA, and Mutant + CMV: TRαA + T3 groups (Figure 4B). These results indicate that *pde6h* promoter is responsive to both TRαA and T3.

## Discussion

During *P. olivaceus* metamorphosis, their lifestyle changes gradually from pelagic to benthic, thereby changing the light environment gradually from brightness to dark with the increasing water depth. As the light environment changes, the visual system of the flounder may undergo adaptive physiological alterations. *PDE6H*, a cone cell-specific inhibitory subunit gene, belongs to the photoreceptor cell-specific PDE6 subfamily (Cote, 2004; Conti and Beavo, 2007). In terrestrial mammals, it can bind to the two domains of PDE6 enzyme, which are a GAF domain and the catalytic domain of the catalytic subunits, and block the activity of PDE6 enzyme in dark conditions (Guo et al., 2006). In light conditions, PDE6 enzyme can be activated in cascade by photon and opsins. Further, the activated PDE6 can effectuate the closure of cyclic nucleotide-gated channels and mediate hyperpolarization of the photoreceptor cell by hydrolyzing cGMP into GMP, finally allowing the light signal to be transmitted (Arshavsky et al., 2002; Zhang et al., 2012; Lagman et al., 2016). In the flounder, the function of *pde6h* gene has not been reported so far.

In this study, we analyzed the expression and regulation of *pde6h* gene by exogenous TH signaling during metamorphosis. The high expression of *pde6h* at the peak of metamorphosis (Figure 1) indicated that it plays an important role in the regulation of flounder metamorphosis. Furthermore, the tissue distribution of *pde6h* gene showed that it is mainly expressed in the eye and hence that it may be an eye-specific gene. By referring to the function of *pde6h* in mammals, we speculated that *pde6h* plays a key role in the development of the eye photosensitive system in the flounder.

Thiourea, an effective TH -depleting drug, has been utilized to study the effects of TH on metamorphosis and can significantly inhibit levels of T4 and T3 *in vivo* during flounder metamorphosis (Davidson et al., 1979; Yu et al., 2017). Previous studies have shown that exogenous TH stimulates metamorphosis of pelagic larvae, producing miniatures of naturally metamorphosed benthic juveniles; in contrast, TU induces metamorphic stasis, resulting in giant pelagic larvae (Inui and Miwa, 1985). Thus, high levels of TH can induce larva to adapt to benthic life earlier. With the completion of metamorphosis, the living environment of flounder changes from pelagic to benthic gradually, and the light environment changes from bright to dark, which might cause physiological changes in the photosensitive system. In this study, we analyzed the levels of *pde6h* mRNA in the TH, NC, and TU groups during larval metamorphosis. These results demonstrate that *pde6h* levels in the TH group larvae are significantly higher than those of the NC group in the early stages of metamorphosis but that they are lower in the TU group during metamorphosis, indicating that *pde6h* gene can be directly or indirectly regulated by TH. The rescue experiment for TU group larvae showed that the metamorphosis-impaired larvae were rescued when TU was removed and that the levels of *pde6h* gene were correspondingly significantly up-regulated in the 41 dph TU + NC and 41 dph TU + TH groups compared to in 36 dph TU and 41 pdh TU groups. So, we conclude that TH can directly or indirectly regulate the expression of *pde6h* gene during flounder metamorphosis.

T4 is rarely active as a pro-hormone of T3; T3 has high activity and performs important physiological functions by acting on its target genes. The promoter regions of the target genes that are regulated by T3 contain thyroid-hormone responsive elements (TREs), which serve as the binding sites for THRs (Cheng et al., 2010; Chiamolera et al., 2012). T3 binding to TRs can result in either a decrease or an increase of the transcription rates of T3 target genes (Oetting and Yen, 2007; Ortiga-Carvalho et al., 2014). TRs can constitutively bind to the TREs of T3 target genes and act in a ligand-independent manner, such that the transcription rate of target genes can change depending on whether or not the THR is bound to T3 (Ortiga-Carvalho et al., 2014). In this study, our dual-luciferase assay indicated that the *pde6h* promoter activity is induced synergistically by T3 and TrαA and that its mutant was not regulated by T3, TRαA, or T3 and TRαA. Therefore, we report that T3 binding to TRαA can promote transcriptional activity of *pde6h* promoter, indicating that *pde6h* is the target gene of T3 in the flounder. However, the specific sites bound by the TH receptors in the six alternative TREs require further investigation.

In summary, we conclude in this study that T3 can directly regulate transcription of *pde6h* gene by binding to TRαA in the flounder, *pde6h* may play an important role in physiological function and eye development during flounder metamorphosis. Furthermore, we speculate that TH may regulate the flounder photosensitive system to adapt to the light changes arising from a transition from pelagic to benthic life during metamorphosis by directly regulating *pde6h* expression. Further study on the function of *pde6h* in the flounder metamorphosis is needed in the future.

## Data Availability Statement

The raw data supporting the conclusions of this article will be made available by the authors, without undue reservation, to any qualified researcher.

## Ethics Statement

This animal study involving fish was approved by the Institutional Animal Care and Use Committee in Shanghai Ocean University.

## Author Contributions

YF designed the study and analyzed the data. YC and JX performed all experiments in this manuscript. NH wrote the manuscript.

## Conflict of Interest

The authors declare that the research was conducted in the absence of any commercial or financial relationships that could be construed as a potential conflict of interest.
